# Time–Temperature Integrating Optical Sensors Based on Gradient Colloidal Crystals

**DOI:** 10.1002/adma.202101948

**Published:** 2021-08-21

**Authors:** Marius Schöttle, Thomas Tran, Tanja Feller, Markus Retsch

**Affiliations:** ^1^ Department of Chemistry Physical Chemistry I University of Bayreuth Universitätsstr. 30 95447 Bayreuth Germany; ^2^ Bavarian Center for Battery Technology (BayBatt) Bavarian Polymer Institute and Bayreuth Center for Colloids and Interfaces University of Bayreuth Universitätsstr. 30 95447 Bayreuth Germany

**Keywords:** binary mixtures, film formation kinetics, infusion withdrawal coating, photonic crystals, structural colors

## Abstract

Manipulation‐free and autonomous recording of temperature states for extended periods of time is of increasing importance for food spoilage and battery safety assessment. An optical readout is preferred for low‐tech visual inspection. Here, a concept for time–temperature integrators based on colloidal crystals is introduced. Two unique features in this class of advanced materials are combined: 1) the film‐formation kinetics can be controlled by orders of magnitude based on mixtures of particles with distinct glass transition temperatures. 2) A gradual variation of the particle mixture along a linear gradient of the colloidal crystal enables local readout. Tailor‐made latex particles of identical size but different glass transition temperatures provide a homogenous photonic stopband. The disappearance of this opalescence is directly related to the local particle ratio and the exposure to a time and temperature combination. This sensing material can be adjusted to report extended intermediate and short excessive temperature events, which makes it specifically suitable for long‐term tracing and threshold applications.

## Graphical abstract

A colloidal stopband‐based time–temperature integrator is introduced, which can monitor the thermal history of a specific system over an adjustable time and temperature range. The fabrication of a binary colloidal crystal gradient comprising same‐sized, monodisperse particles with distinct glass transition temperatures are the key components for this type of integrating sensor.

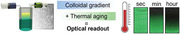

## 1 Introduction

Particle‐based colloidal crystals (CCs) and inverse opals have been subject to extensive research for a long time.^[^ ^1^ ^]^ For homogeneous and patterned colloidal assembly structures a wide range of fabrication techniques has been investigated and established, which render such nanostructured films a mature area of research.^[^ ^2^ ^]^ The intricate nanostructure on a length scale of a few to hundreds of nanometers raised interest in fields such as granular mechanics,^[^ ^3^, ^4^ ^]^ heat transport,^[^ ^5^, ^6^ ^]^ phononics,^[^ ^7^ ^]^ and catalysis,^[^ ^8^, ^9^ ^]^ to name a few. By far most prominent is, however, their periodic refractive index modulation, resulting in vivid structural colors. Consequently, colloidal crystals are predestined for sensing, where significant color changes can often be recognized with the bare eye.^[^ ^10^, ^11^, ^12^ ^]^ A shift of the optical stopband, as well as a change of the opalescence intensity, can serve as an indicator for changes inflicted to the colloidal ensemble. Such changes can be caused by temperature,^[^ ^13^, ^14^ ^]^ force,^[^ ^15^, ^16^ ^]^ humidity,^[^ ^17^, ^18^, ^19^ ^]^ pH,^[^ ^20^, ^21^ ^]^ ionic strength/complexation,^[^ ^22^ ^]^ wettability,^[^ ^23^, ^24^ ^]^ or biodegradation^[^ ^25^ ^]^ and either alter the refractive index contrast (change in intensity), the periodicity of the structure (change in bandgap), or both. The sensing performance can be further tuned, for example, by the introduction of fluorescence for organic vapor detection.^[^ ^26^, ^27^, ^28^ ^]^

An important and general distinction of sensors is their classification into reversible and irreversible ones. Reversible sensors indicate the actual condition of a system in real‐time. Temperature‐dependent color changes of liquid crystals are a wide‐spread example of this. In the case of monitoring certain predefined limits, irreversible sensors are more suitable as they allow judging the history of a certain state. Irreversible sensors are especially relevant in food or drug transportation and storage.^[^ ^29^, ^30^ ^]^ When the readout response changes in a gradual and slow manner, irreversible sensors can also indicate the degree to which a certain state has been exceeded. This provides additional information either regarding the time or intensity that a certain state has been reached. Considering the role of colloidal crystals in the field of irreversible sensors, the loss of opalescence at the film‐forming temperature is obvious. This process is also called dry‐sintering and has been studied already.^[^ ^31^, ^32^, ^33^ ^]^ The onset of dry‐sintering is related to the glass transition temperature (*T*_g_) in the case of polymer colloidal crystals. At this point, the optical and thermal properties change abruptly, corresponding to the structural degradation process. The concomitant loss of contrast and periodicity diminishes both photonic opalescence and thermal insulation properties.^[^ ^34^, ^35^ ^]^ Introduction of additives can alter the kinetics and reversibility of this film formation process, which allows correlating time and temperature processes.^[^ ^36^ ^]^ Random mixtures of two monodisperse particle types with different glass transition temperatures allowed to change the abrupt jump in thermal conductivity at the glass transition temperature to a gradual one.^[^ ^37^ ^]^ An even more elaborate microstructural design with locally controlled film formation kinetics was introduced by Lee et al.^[^ ^38^ ^]^ Polymer inverse opals with locally varied cross‐linking densities allowed to determine the temperature and exposure time simultaneously.

The aforementioned work is an excellent example of the emerging possibilities of future functional materials, where unconventional properties can be realized by a local control on the fundamental material properties. Lithographic (micro)patterning of colloidal structures has been investigated by various groups already.^[^ ^39^ ^]^ Much fewer systems have been reported in the case of CCs and inverse opals, where the structure or composition is gradually altered. First examples for tuning the lattice spacing have been realized by means of diffusion,^[^ ^40^, ^41^ ^]^ compression,^[^ ^42^, ^43^ ^]^ wrinkling,^[^ ^44^, ^45^ ^]^ or an external magnetic field.^[^ ^46^ ^]^ Ultracentrifugation has also been used for the preparation of colloidal gradients.^[^ ^47^, ^48^, ^49^ ^]^

In this work, we demonstrate how a controlled spatial variation of the colloidal crystal composition allows realizing a time–temperature integrator. We introduce two new aspects to the field of colloidal materials. First, we elaborate a logarithmic dry‐sintering kinetic behavior based on random binary CCs consisting of high‐ and low‐*T*_g_ polymer particles. Second, we introduce a method to fabricate thin‐film colloidal crystals with a compositional gradient. The synergistic combination of composition‐dependent kinetics and local composition control results in an adjustable time–temperature integrating sensor with a position‐dependent optical readout.

## 2 Results and Discussion

**Figure 1:**
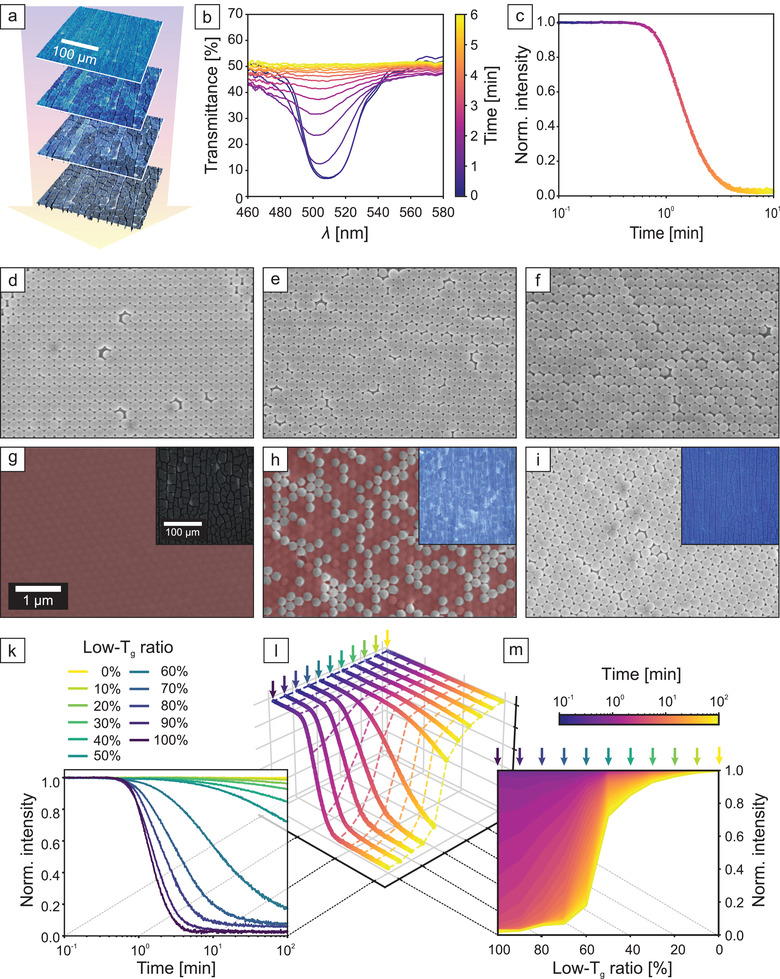
Characterization of the film‐formation of colloidal crystals at 60 °C. a) Optical microscopy images show the simultaneous color fading (blue to gray) and crack formation (thin dark lines) during the film‐formation of 100% low‐*T*_g_ particles. b) In situ transmission UV–vis spectra showing the temporal change of the photonic stopband during the film‐formation process shown in (a). c) Time‐dependent decay curve calculated from the normalized stopband intensity shown in (b) to quantify the loss of opalescence. d–f) Scanning electron microscopy images of pristine colloidal crystals consisting of 100%, 50%, and 0% low‐*T*_g_ particles, respectively. g–i) Images of the same samples after 100 min at 60 °C. Dense, sintered regions are color‐coded in red, original SEM images are shown in Figure S4, Supporting Information. Insets show light microscopy images, where a loss of the photonic stopband and the appearance of cracks is visible when transitioning from a pristine to a sintered film. k) Stopband decay curves of various compositions between 100% and 0% low‐*T*_g_ particles. (l) and (m) show how the data in (k) can be transformed to convert the time‐dependent measurement into a composition‐dependent analysis.

The dry‐sintering kinetic properties of a homogeneous CC consisting of purely low‐*T*_g_ particles are well known (**Figure** **1**a). We, therefore, fabricated homogeneous colloidal crystals comprising monodisperse poly[(methyl methacrylate)‐*random*‐(butyl acrylate)] (PMMA‐*r*‐nBA) colloids via dip‐coating from aqueous dispersions. As a larger contact area between neighboring particles is formed and the air is expelled from the structure, the periodic variation of the refractive index is lost. Concomitantly, the structural color vanishes and the cracks between crystalline domains become larger to accommodate shrinkage, which can be seen in the microscopy images from top to bottom (Figure 1a). Transmission UV–vis spectra (Figure 1b) show a slight blueshift of the photonic stopband of approximately 10 nm and an even more pronounced decrease of the intensity (purple to yellow color). The normalized stopband intensity can be used as a measure for the kinetics of the film formation process (Figure 1c, details in Figures S1 and S2, Supporting Information). We use a semi‐logarithmic scale for a clearer representation of the temporal evolution of the sintering behavior. For the specific case shown here (*T*_g_ = 49 °C, sintering temperature *T* = 60 °C), the film formation process is complete after about 4 min (normalized intensity decayed to ≈0). Scanning electron microscopy (SEM) images of the colloidal crystal before and after sintering at 60 °C for 100 min (Figure 1d,g) confirm the expected loss of porosity during the film formation.

Adding various amounts of a second latex bead of the same size but with a different glass transition temperature (*T*_g_ = 94 °C, Figure S3, Supporting Information) to the dip‐coating dispersion does not compromise the colloidal crystal formation, but it strongly influences the film formation process. When 50% high‐*T*_g_ particles are added, the hexagonal lattice structure is retained, but only half of the particles experience deformation during sintering at 60 °C (Figure 1e,h). This leads to particle patches with retained shape and interstitial space. The surface impression provided by SEM is confirmed by the bulk behavior of the stopband, where a blue structural color is retained (Figure 1h, inset). Heating a CC composed purely of high‐*T*_g_ particles at 60 °C shows no effect, and the structure remains unaltered (Figure 1f,i).

Analogous to the pure low‐*T*_g_ system, we quantified the sintering kinetics of various binary CC compositions by in situ UV–vis measurements at 60 °C. Figure 1k summarizes the time‐dependent decay of the normalized stopband intensity for these systems. Quite strikingly, the binary compositions shift the film formation kinetics by orders of magnitude to longer time scales with an increasing amount of high‐*T*_g_ particles. Each individual system follows an exponential decay at first glance. We, nevertheless, want to stress that this optical analysis is not sufficient to unravel the detailed mechanics of this process. Dry‐sintering of polymer colloids takes place in a number of different steps, including, for example, contact area formation.^[^ ^32^ ^]^ The underlying refractive index matching is presumed to arise from viscous polymer flow of the low‐*T*_g_ component and capillary infiltration of cavities between non‐sintered particles. Each step most likely shows a unique temperature dependence, and we will therefore compare these results only qualitatively. One may infer a jump in decay kinetics between the 50% and 60% low‐*T*_g_ particle composition in Figure 1k. We rationalize this by examining SEM images of all sample compositions sintered at 60 °C (Figure S4, Supporting Information). A gradual change in composition results in a transition from a continuous low‐*T*_g_ particle network to isolated domains. This is most evident between 40–60% low‐*T*_g_ particles, where the majority phase inverts. The presence of islands of one particle type is not an effect of phase separation. In Figure S5, Supporting Information, SEM images of partially sintered CCs are compared to a 2D‐simulation of a random distribution of two particle types. An analysis of the mean absolute number of neighboring, non‐sintered particles in the first, second, and third generation is performed for both the measured and simulated images. The relative intensities as well as the absolute values show a significant overlap between the experimental and simulated data. This comparison confirms the statistical distribution expected due to the same size and surface chemistry of the two particle types.

The observed sintering behavior is rather intriguing as it allows an adjustment of the thermal response simply by mixing particles at a defined ratio with two distinct glass transition temperatures. The dependence of time, composition, and normalized stopband intensity result in a wide parameter space that can be difficult to grasp. We, therefore, introduce an alternative way to visualize the film formation kinetics (Figure 1l,m). Here, the normalized intensity is plotted versus the composition and color‐coded with respect to the sintering time. Each line represents one specific composition. Figure 1l connects the time‐scale to the crystal composition. It also helps to understand the representation in Figure 1m, from which the expected stopband intensity can be derived for any sintering time and any particle composition. For the sake of clarity, we point out to the reader that throughout this work, the color scheme shown in Figure 1k (yellow to turquois) corresponds to a change in composition, while a time dependency is indicated by the color scheme in Figure 1m (yellow to purple).

**Figure 2:**
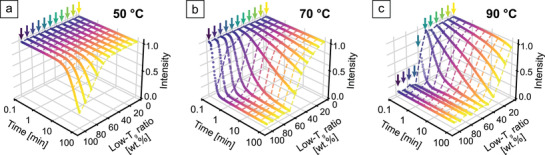
Three‐dimensional representation of stopband decay curves. Several compositions are measured at temperatures of 50 °C (a), 70 °C (b), and 90 °C (c). The dashed lines are guides to the eye. They represent the composition‐dependent intensity profile expected for the sintering behavior after certain time steps.

Further in situ measurements of the various particle ratios are conducted at temperatures of 50, 70, and 90 °C (**Figure** **2**a–c). As 50 °C is close to the lower glass transition temperature, the observable effect is comparatively small. None of the samples showed complete thermal degradation during 100 min of measurement time. A gradual change from 100–50% low‐*T*_g_ particles is present beyond which none of the curves exhibit a discernible decay. Increasing the temperature to 70 °C causes the low‐*T*_g_ dominated samples to degrade in a matter of seconds to minutes. The perceptible change of the profile shape at longer sintering times is shifted to the range between 70–20%. This is amplified when the temperature is raised further to 90 °C. Here, a slight degradation even of the pure high‐*T*_g_ particles sets in. In total, the temperature and time‐dependent behavior shows how binary CCs could be used as time–temperature integrators. The combination of various compositions and their normalized intensities correlate to certain combinations of the sintering time at a certain temperature. Limits of this particular system are given by a lower temperature where no effect is observed at approximately 45 °C and an excessively fast film formation of all particles above 100°C (Figure S6, Supporting Information).

**Figure 3:**
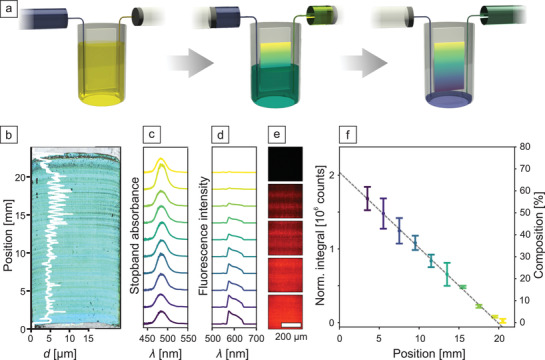
Infusion withdrawal coating technique to fabricate gradient colloidal crystals. a) Simultaneous infusion and extraction of the colloidal dispersion at different rates leads to a continuous composition gradient. This gradient is coated on the submerged glass substrate by continuous extraction of the mixed dispersion. b) Stitched light microscopy image of a colloidal crystal prepared via infusion withdrawal coating. The overlay (white line) shows the thickness of the colloidal film measured along the coating axis. This sample comprises a gradient of fluorescently labeled tracer particles. c,d) Stopband absorbance and fluorescence intensity at various gradient positions, respectively. e) Example fluorescence microscopy images taken along the gradient. f) Change of the normalized fluorescence intensity versus gradient position. A linear profile of the change in composition is obtained. The error bars result from measurements on three individual samples.

To fully exploit the potential of this composition‐dependent film formation behavior, we demonstrate now how to arrange the binary particles in a gradient colloidal crystal. For this, we took inspiration from infusion‐withdrawal‐coating (IWC), which has been reported for sol–gel‐derived gradients.^[^ ^50^, ^51^, ^52^, ^53^ ^]^ This method is based on dip‐coating by the use of two syringe pumps (**Figure** **3**a). A glass substrate is dipped in a dispersion of one particle type, which is extracted with a syringe pump. Simultaneously, a second syringe pump infuses a dispersion of the second particles at a slower rate. The water level, thereby, decreases continuously, mimicking dip‐coating, while the composition changes in a slow and gradual fashion. This time‐dependent concentration change translates directly into a compositional gradient along the coated substrate. Similar to dip‐coating, this produces a continuous thin‐film with a strong iridescent color throughout the entire sample. Figure 3b also shows a typical colloidal crystal with a film thickness between 4–8 µm. The periodic roughness, especially on the top half of the sample, is a consequence of meniscus pinning and stick‐slip behavior.^[^ ^54^ ^]^ Transmission UV–vis spectra in Figure 3c measured at equidistant points along the coating axis corroborate the successful colloidal assembly. The stopband of this photonic crystal is distinct at all points. Peak intensities vary slightly, which is attributed to the observed thickness modulation. Peak position and FWHM by and large remain constant, emphasizing the homogeneity and high quality of the gradient colloidal crystal. To visualize and quantify the gradual composition change, we introduced red‐fluorescent polystyrene particles to the infusion dispersion. These fluorescent tracer beads (100 nm diameter) are small enough to theoretically occupy octahedral gaps in the structure and were added in a trace amount of 1 wt% with respect to the PMMA/nBA particles.

Fluorescence microspectroscopy (Figure 3d,e) confirms the targeted composition gradient. A gradual increase of the fluorescence intensity is spectroscopically measured and corroborated by microscopy images exhibiting a transition from black to red. Modulation of the layer thickness can also be observed in the microscopy images. The fluorescence intensity is expected to be proportional to the number of tracer particles and to the film thickness. Quantification of the gradient is, therefore, performed by integration of the fluorescence signal between 560–650 nm. The integrated fluorescence is then corrected to the local layer thickness by normalization to the stopband intensity measured at the same position. The validity of this analytic procedure is assessed by measuring homogeneously dip‐coated samples with defined tracer particle concentrations between 0–1.0 wt% (Figure S7, Supporting Information). The expected linear trend of the tracer particle concentration could be reconstructed when normalizing to the stopband intensity. This analysis confirms the direct relation between fluorescence particle concentration and fluorescence intensity. We use this direct relationship to measure the normalized fluorescence integral at identical positions along three individual gradient samples. Figure 3f shows that the composition profile follows a linear trend. Despite the homogeneous photonic stopband of this binary system, we were able to prepare a linear gradient of the tracer particle incorporation. Due to some limitations inherent to the IWC setup, we can access gradients ranging from 0–60% composition.

**Figure 4:**
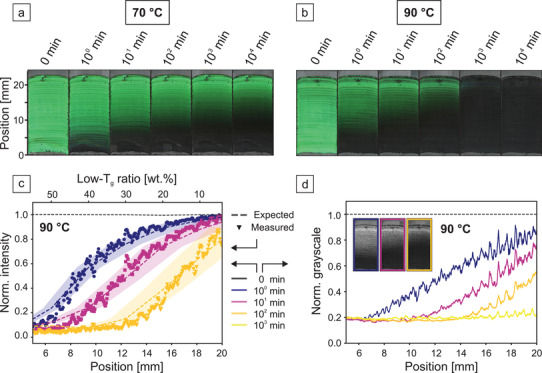
Colloidal crystals with a gradual change of the ratio of high‐ and low‐*T*_g_ particles. (a) and (b) show identically prepared gradients and the sintering procedure at 70 and 90 °C, respectively. Images are obtained via stitching of light microscopy images and ex situ during the sintering process. The respective sintering times increase on a logarithmic scale. c) Normalized intensity obtained from ex situ UV–vis transmission measurements along the gradient axis. The dotted lines correspond to expected profiles originating from in situ measurements shown in Figure 2c. The shaded areas arise from the errors in the linear fit in Figure 3f, which is needed to correlate distance and composition. d) The inset shows green‐channel images after color‐channel separation of the pictures in (b). Profile analysis normalized to the measurement of the pristine gradient shows analogous results to the ex situ UV–vis measurements.

Using this approach, we prepared CCs with a gradually changing ratio of high‐ and low‐*T*_g_ particles. Two identical gradients are subjected to sintering temperatures of 70 and 90 °C, respectively (**Figure** **4**a,b). In both cases, dry‐sintering and loss of structural color sets in first at the bottom of the sample where the amount of low‐*T*_g_ particles is highest. While the temperature persists, the threshold between the colorful and degraded regions moves along the gradient. As expected, this film‐forming process proceeds faster at a higher temperature. To better quantify these observations, we evaluate the normalized peak intensity along the gradient sintered at 90 °C (Figure 3c). This data is obtained from ex situ UV–vis measurements (Figure S8, Supporting Information) after the gradient structure has been subjected to the respective time and temperature. The stopband decay correlates well with the aforementioned observations. It becomes especially clear how sintering times on a logarithmic scale can be distinguished. The profiles at 1 and 10 min are just as well separated as those of 10 and 100 min. We further corroborate the reproducibility of the kinetic behavior by including the expected composition‐dependent intensity decay derived from Figure 2c as dotted lines in Figure 4c. This is possible by translating the position along the gradient into a low‐*T*_g_ ratio via the results from the fluorescence measurements. Expected and measured data overlap quite well and substantiate the controlled and predictable sintering behavior of the nanostructure. Measurements along the same gradient heated to 70 °C in Figure S9, Supporting Information, show a profile where dry‐sintering is primarily observed at the bottom of the sample where more low‐*T*_g_ particles are present. This is corroborated by SEM images of the partially sintered gradient (Figure S10, Supporting Information). In this case, distinguishing sintering times between 1, 10, and 100, and even 10^3^ and 10^4^ min is possible. Further examination shows that similar intensity profiles can be reached by various time–temperature combinations. For the case shown here, annealing at 70 °C for 100 min and 90 °C for 1 min result in comparable measurements. This behavior portrays the expected sensitivity to both temperature and time. We point out that a correction coefficient is applied to the expected data to shift the axis of the particle composition relative to the position on the substrate in Figure 4c. This is necessary due to subtle and non‐systematic variations during the individual coating processes. Such variations are likely caused by inconsistent pinning of the meniscus, variations of the substrate wettability, and the relative humidity during coating. The correction is done by optimizing a shift parameter to overlap the expected and measured data at a sintering time of 1 min. In the case of the 90 °C measurement, the axis is shifted by 0.8 mm. This is a reasonably small variability given the dynamic position of the wetting meniscus. Profiles of ensuing measurements at longer sintering times then all show a good agreement between the expected and measured intensity profiles.

The simplest method of analyzing the sintering behavior is the observation of the structural color at different degrees of sintering. The sintering difference along the composition gradient is obvious to the bare eye (Figure 4a,b). Simple image analysis is sufficient to obtain a more quantitative analysis, which reaches a comparable significance compared to the spectral analysis shown in Figure 4c. The separation of the color channels and profile analysis of the green channel image is shown in Figure 4d. The distinction of sintering times on the same logarithmic time‐scale is clearly visible. To provide a benchmark, the accuracy of temperature and time determination by green‐channel evaluation is provided in Figures S11 and S12, Supporting Information, respectively. These experiments show that, depending on the absolute temperature region, temperature steps between 5–10 °C can clearly be distinguished. Regarding the temporal evaluation, approximately 1–2 steps can be resolved between each decade. Despite the uniquely simple measurement and evaluation, reproducibility is not impaired as shown in Figure S13, Supporting Information. Identically prepared colloidal crystal gradients show green‐channel profiles with a strong overlap when subjected to the same thermal history.

This method of evaluation requires no elaborate equipment and works with a low‐level software realization. We expect that given correct lighting conditions and a reference region on each sample, this analysis could be readily achieved by modern smartphones. This provides the context for a potential application of such gradient‐based time–temperature integrators. These structures operate autonomously and cannot be restored nor refreshed once the film formation sets in and are, therefore, not susceptible to manipulation. The distinct property of this gradient sensor is its sensitivity to prolonged moderate temperature exposures and short high‐temperature excesses. The temperature can be imposed by the environmental conditions or caused by an operating device serving as heat source. Hence, a continuous monitoring of, for example, the thermal history of high‐power batteries is readily conceivable, where heat management is gaining increased attention. High operating temperatures have pronounced negative effects regarding, for example, capacity fading.^[^ ^55^, ^56^ ^]^ Batteries that experience excessive temperatures can additionally become a safety hazard.^[^ ^57^ ^]^ As batteries are an ubiquitous part of everyday life, it is of great importance to make an assessment of the thermal history as simple as possible. The colloidal gradients introduced here provide a visual way to judge on the time–temperature history based on the position‐dependent stopband intensity. Beyond a certain threshold of local film formation, either the desired life expectancy would have been surpassed, or an undesired high‐temperature excursion appeared. In either case, a thorough analysis of the battery state would be required before further usage. For such potential applications the current model system needs to be improved by a specific design of the required sensitive temperature range. This can be achieved by fine‐tuning the composition range, by realizing non‐linear composition gradients, and by creating gradient strips with particles of different, pre‐defined *T*_g_.

## 3 Conclusion

We have demonstrated a novel approach to functional colloidal crystals, which can be used as time–temperature integrators. Two key advancements in colloidal material fabrication made this sensor based on structural color detection possible. First, mixing of two equally sized, monodisperse latex particles with distinct glass transition temperatures provides access to composite colloidal crystals with specific time‐dependent film formation properties. The film formation kinetics at a certain temperature can be varied from a few seconds to hours, days, and weeks by choosing the right particle composition. Second, the fabrication of gradient colloidal crystals allows the translation of the particle composition to a local position. We, therefore, introduced infusion‐withdrawal coating for the fabrication of linear composition gradients at a retained optical stopband. The gradient structures exhibit the expected film‐formation kinetic behavior and, consequently, change the intensity of the optical stopband locally. The gradual and local transition can be analyzed spectroscopically or through simple image analysis and provides a simple, autonomous, and manipulation‐free way to assess the colloidal gradient's thermal history. Our contribution demonstrates how space‐specific material engineering allows fabricating structures that exceed their individual components′ properties.

## 4 Experimental Section

Details on the particle synthesis, coating procedures, and characterization methods, as well as details regarding the data evaluation of UV–vis spectroscopy, fluorescence spectroscopy, and scanning electron microscopy images can be found in the Supporting Information.

## Conflict of Interest

The authors declare no conflict of interest.

## Supporting information

Supporting Information

## Data Availability

The data that supports the findings of this study are available in the supplementary material of this article.
