# Supplementary material for: Time–Temperature Integrating Optical Sensors Based on Gradient Colloidal Crystals
Source: Adv Mater. 2021 Aug 21;33(40):2101948. doi: 10.1002/adma.202101948 (PMC11468944; doi:10.1002/adma.202101948)
Supplement: Supplementary file 1 — Supporting Information [file ADMA-33-2101948-s001.pdf]

© 2021 Wiley-VCH GmbH

ADVANCED MATERIALS

Supporting Information

for *Adv. Mater.,* DOI: 10.1002/adma.202101948

Time–Temperature Integrating Optical Sensors Based on Gradient Colloidal Crystals

*Marius Schöttle, Thomas Tran, Tanja Feller, and Markus Retsch**

Supporting Information

Time-temperature integrating optical sensors based on gradient colloidal crystals

*Marius Schöttle[1], Thomas Tran[1], Tanja Feller[1], and Markus Retsch[1,2,]**

Experimental Section

Materials:

Methyl methacrylate (MMA), n-butyl acrylate (nBA), 3-styrenesulfonic acid sodium salt hydrate (NaSS, ≥99%), and potassium persulfate (KPS, ≥99%) were obtained from Sigma-Aldrich. MMA and nBA are destabilized over Alox B prior to use. Sulfate-modified, fluorescent red polystyrene particles (100 nm diameter) were obtained from Sigma-Aldrich as an aqueous suspension, diluted to 0.025 wt.%, and sonicated for 20 minutes before further use. Water is taken from a Millipore Direct Q3UV unit for all experiments.

Particle synthesis: Monodisperse polymer colloids are prepared via an emulsifier free emulsion polymerization. 13 mL monomer mixture (MMA/nBA, either 70:30 or 90:10) are added to 250 mL water and heated to 80 °C. After stirring at 850 rpm for 60 minutes under nitrogen flow, 10 mg NaSS and subsequently 100 mg KPS, both dissolved in 5 mL water, are added quickly. After 15 minutes, the stirring speed is reduced to 650 rpm. The reaction is left to proceed over-night and terminated by exposure to ambient oxygen.

Substrates: Glass substrates are cleaned carefully prior to all coating procedures. Ultrasonication proceeds twice in 2 vol.% aqueous Hellmanex III solution and once in ethanol (p.a.). Subsequently, substrates are immediately dried under a nitrogen stream. Directly before coating, the surface is hydrophilized via oxygen plasma treatment.

Dip-coating: Homogeneous colloidal crystals are prepared via dip-coating. Several dispersions with varying ratios of particle types are prepared. Mixtures are adjusted to 1.0 total wt% and stirred over-night to ensure statistical distribution of the two particle types.

Clean glass substrates are inserted, and dip-coating proceeds with a velocity of 0.25 µm s^{-1} and a controlled atmosphere of 25 °C and 75% relative humidity.

Infusion-withdrawal-coating: Gradient colloidal crystals are prepared via an infusion-withdrawal-coating process. A clean glass substrate is dipped in a 10 mL PTFE-beaker filled with 8.7 mL of the starting dispersion and equipped with a stirrer bar. The stirring speed is set to 80 rpm. Two cannulas are inserted vertically on opposite sides of the beaker, and each is connected to a syringe pump. One injects the second dispersion at 0.6 mLh^{-1}, the other extracts the resulting mixture at 1.1 mLh^{-1}. Taking into account a determined evaporation rate of 0.1 mLh^{-1}, this ensures that the extraction rate is twice the infusion rate. The first part of the colloidal crystal is especially subject to pinning and defect formation. Therefore, before the infusion is started, extraction is set to 0.5 mLh^{-1} for one hour. The corresponding top part of the sample is not regarded in the evaluation. The concentration of both dispersions is 1.3 wt%, and the ambient conditions lie between 19 - 21 °C and 25 - 35% relative humidity. For the fluorescence measurements, one PMMA/nBA type is used, and 1.0 wt.% (with respect to PMMA/nBA) red-fluorescent polystyrene particles are added to the infusion dispersion.

Methods:

Differential scanning calorimetry: Measurements are conducted using a TA Instruments Discovery DSC 2500. The second of two heating cycles is used for the evaluation. Samples are measured between -20 – 200 °C at 10 K min^{-1} and in a nitrogen atmosphere.

Imaging microscopy: 2D color images and 3D reconstructed images are obtained using a laser scanning microscope (Olympus, LEXT). High magnification images are taken using a 50x lens with N.A. 0.95. Overview images are obtained by stitching several domains with a 5x lens with N.A. 0.15.

UV-Vis/Fluorescence microspectroscopy: The setup is based on an Olympus IX71 inverted microscope. Throughout all measurements, a 4x lens with N.A. 0.10 is used. UV-Vis spectra are obtained in transmission geometry with a halogen light source. An OceanOptics USB4000

spectrometer is coupled via fiber optics. *In-situ* measurements are conducted by using an Instec HCS622HV heating stage with a silver heating block and transmission capability to the setup. The lid of the stage is lifted briefly while the sample is placed on the preheated silver block, and the measurement is immediately started. Spectra are obtained every 500 ms over a period of 100 minutes. Ex-situ measurements are performed by externally heating samples on the same heating stage for a defined amount of time. Subsequently, a motorized µm-stage is used to automatically measure spectra at defined positions along the samples.

Fluorescence spectroscopy is performed with the same setup but with a mercury vapor lamp in reflection geometry. A Chroma 49005 Cy3 filter cube is used, allowing excitation between 530 - 560 nm and emission detection between 590 - 650 nm. The integration time is set to 2 seconds, and 10 scans are measured for averaging. Gradients are measured at defined positions using a µm-stage. Thereby, fluorescence and transmission UV-Vis spectra can be measured at the same position.

Scanning electron microscopy: Images were taken with a Zeiss Ultra plus (Carl Zeiss AG, Germany) at an operating voltage of 3 kV and with in-lens detection after sputtering of 2 - 4 nm platinum.

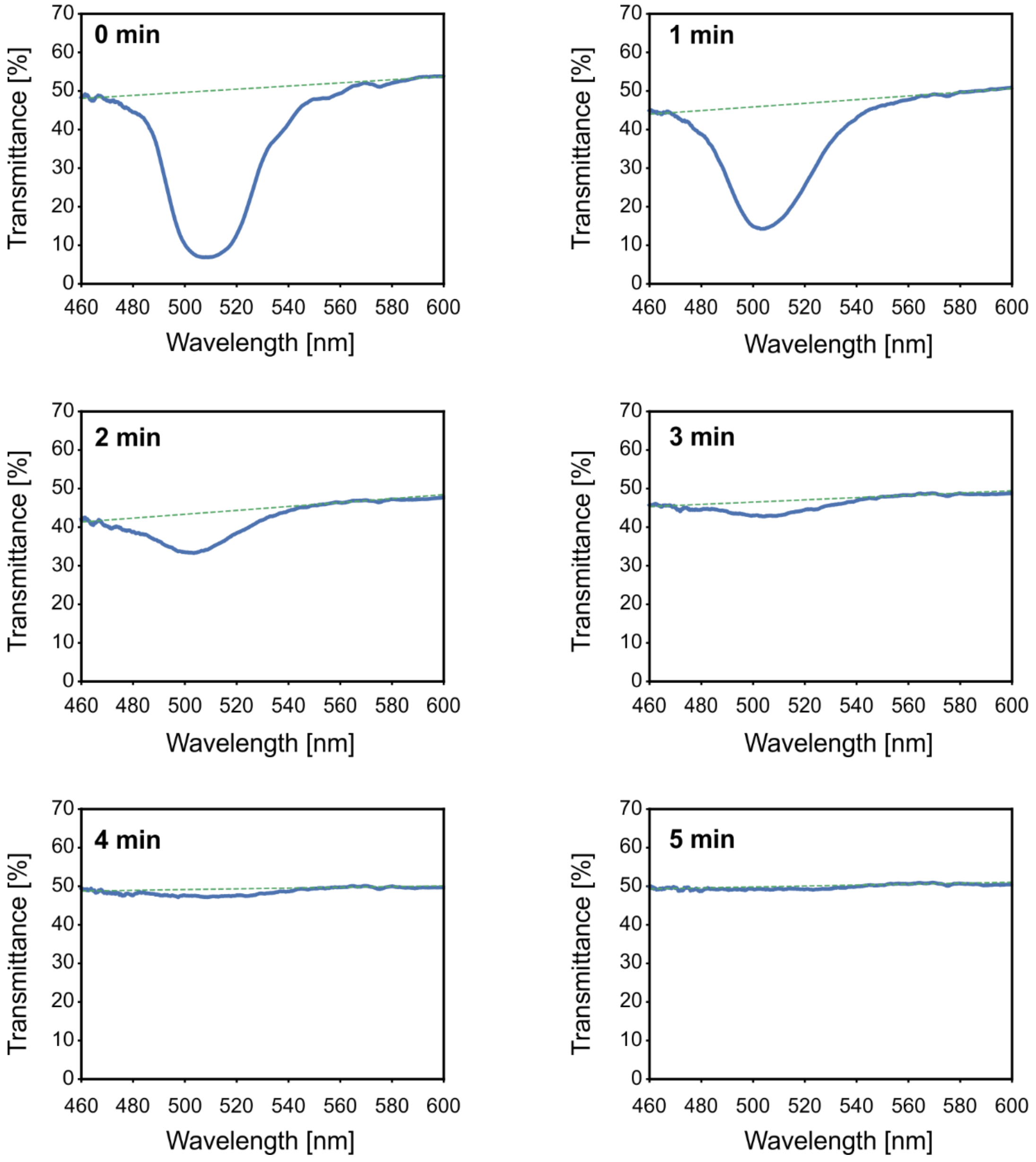

Figure S1. Transmission UV-Vis spectra during the film-formation of a 100% low-T_g colloidal crystal at 60 °C. Dotted green lines show the baseline used for the evaluation. Division of the spectrum by the baseline and subsequent normalization to the stopband intensity at 0 minutes leads to the decay curve in **Figure 1c**.

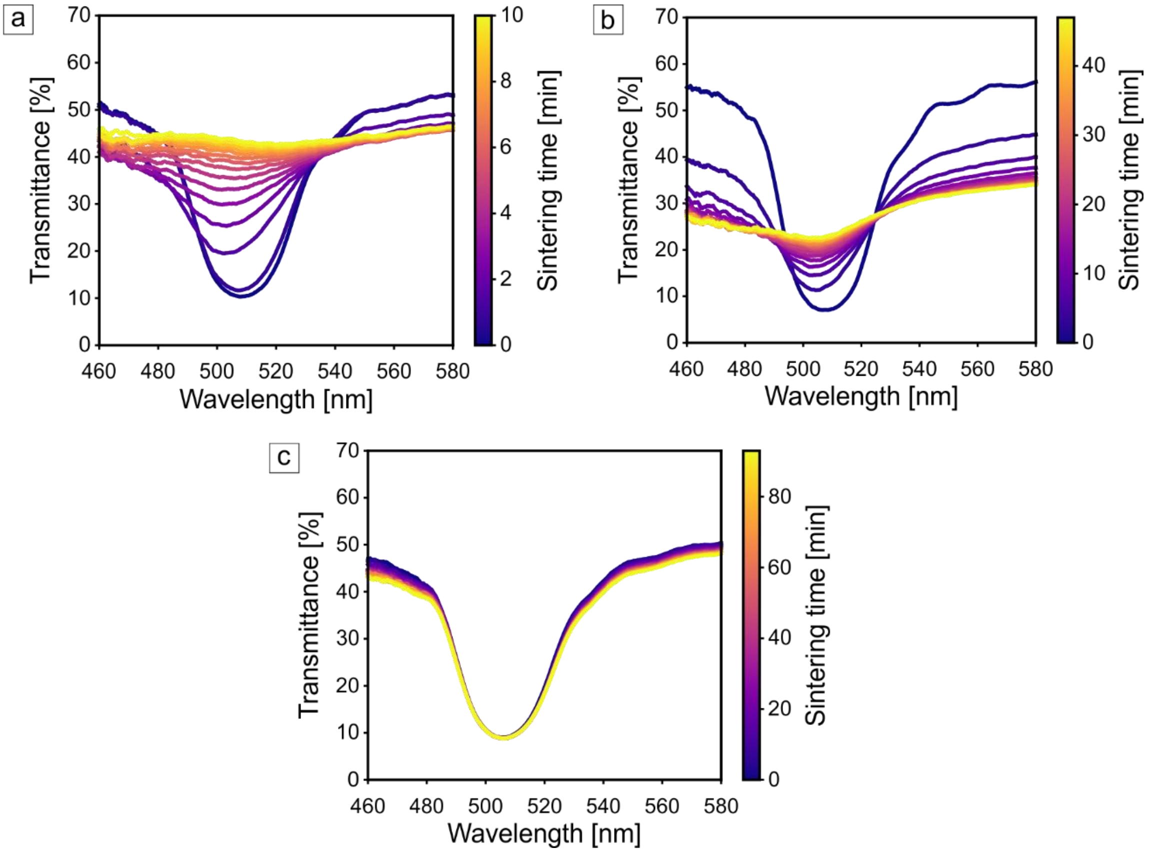

Figure S2. Exemplary *in-situ* UV-Vis measurements of samples with a) 80, b) 60, and c) 10% low-T_g particles at 60°C. After 5 minutes, the spectra in a) show slight minima and maxima that arise from thin-film interference. This cannot accurately be distinguished from the stopband and is therefore not corrected. As an effect, the normalized intensity decays to a value slightly above zero.

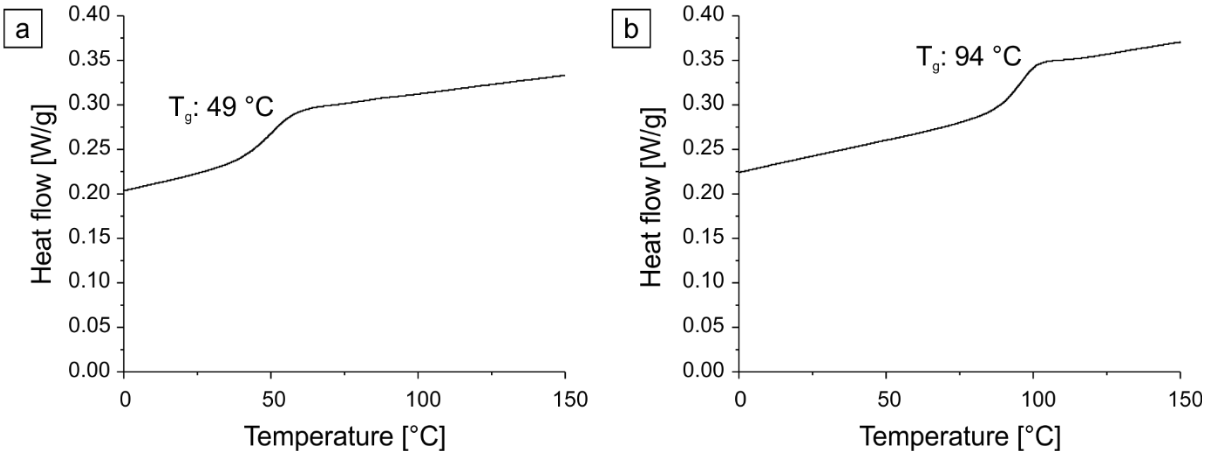

Figure S3. The second heating curve of DSC measurements of PMMA/nBA particles with a monomer ratio of a) 70:30 and b) 90:10.

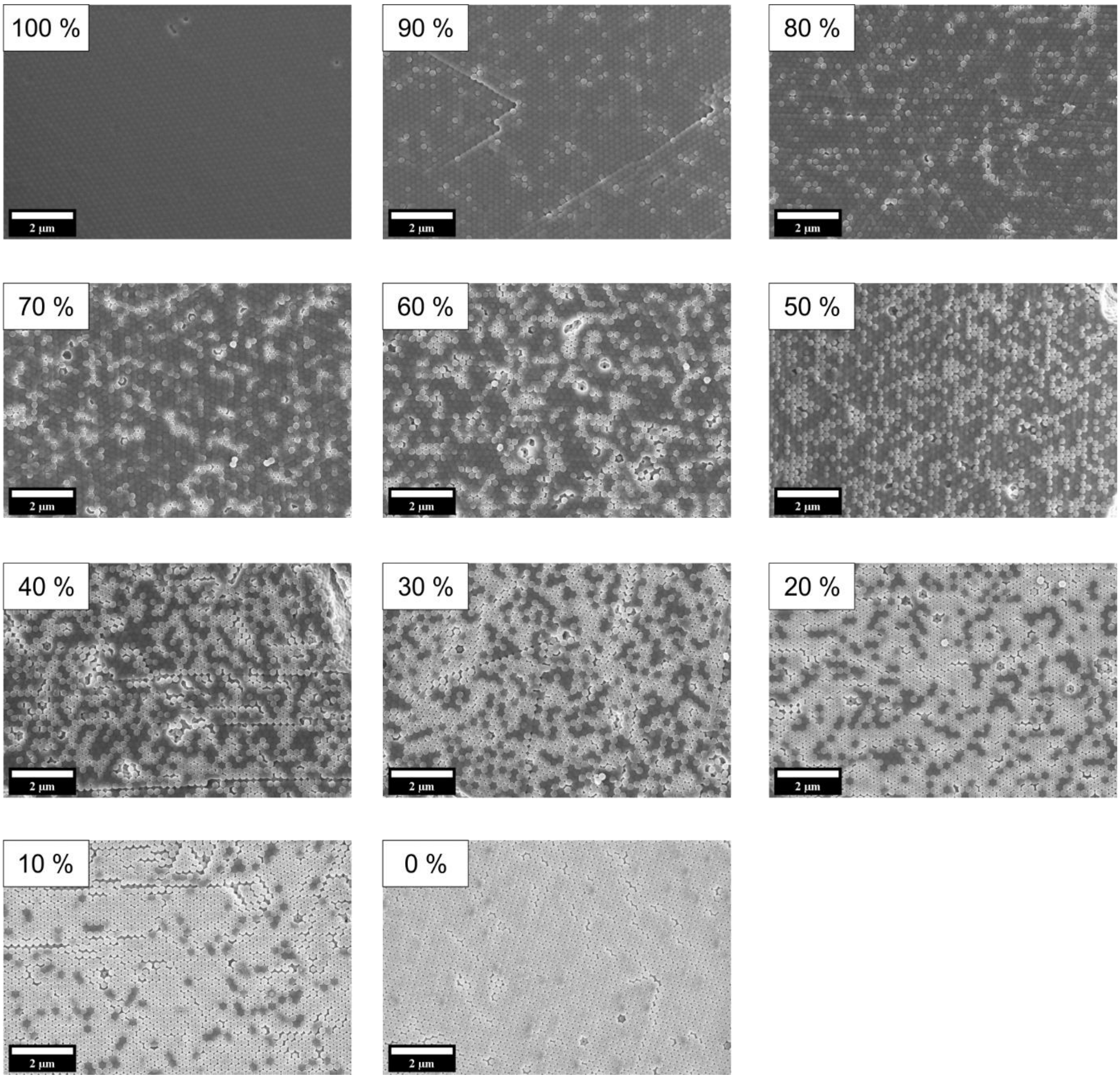

Figure S4. Scanning electron microscopy images of colloidal crystals after film formation for 100 minutes at 60 °C. Different compositions are labeled with the amount of low-T_g particles in the mixture.

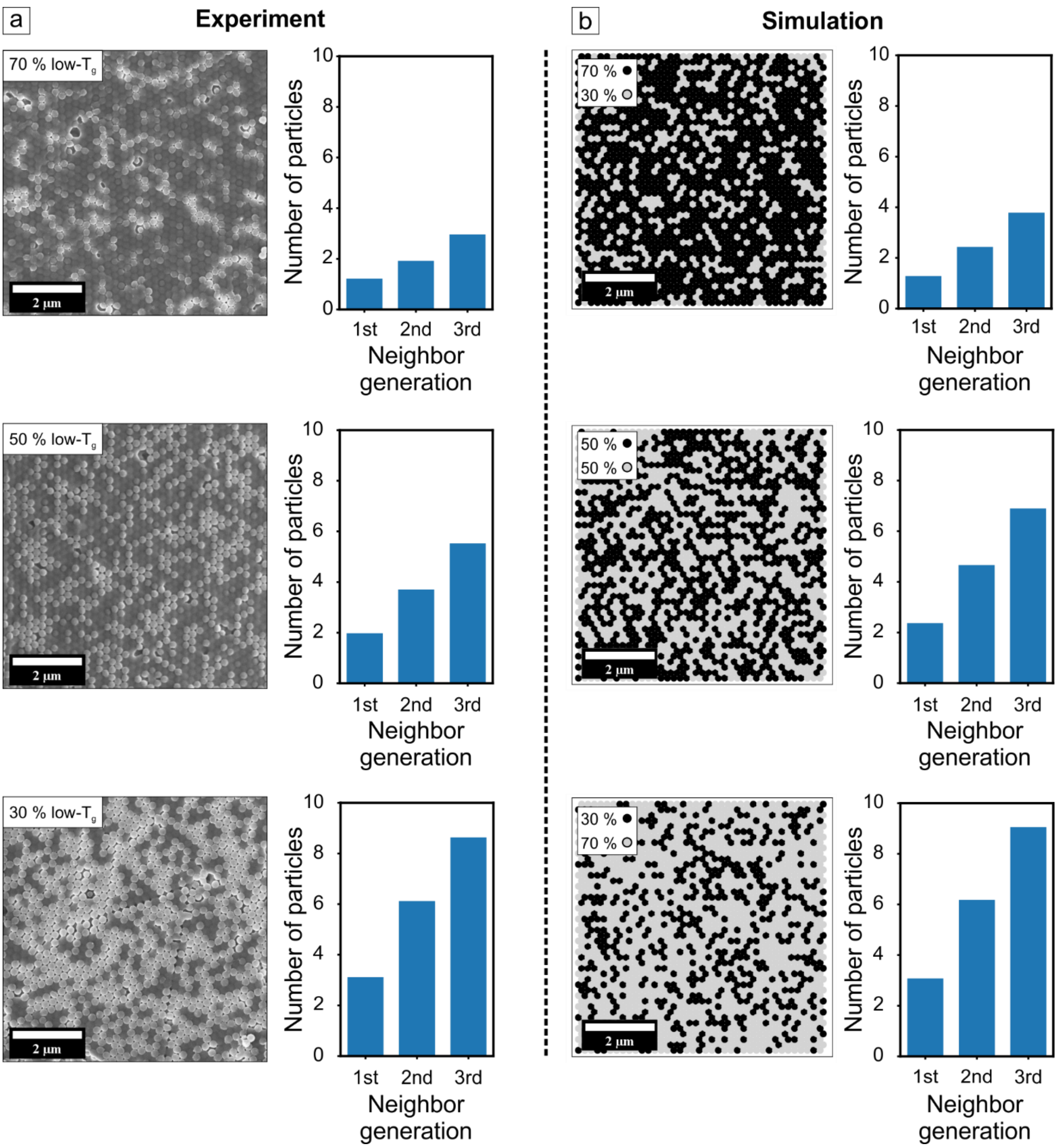

Figure S5. a) Scanning electron microscopy images of colloidal crystals with a different amount of low-T_g particles after film formation. b) Simulated distributions of two different particle types randomly mixed in a 2D-hexagonal structure. The images are calculated by randomly assigning each particle with a black or grey color. The probability of each color is chosen according to the particle ratio in the SEM counterpart.

For the quantitative evaluation, the positions of non-sintered particles in the SEM images and grey particles in the simulations are examined. The mean number of neighbors is evaluated in both cases. First generation neighbors are integrated between 0 - 0.3 µm distance between particle centers, second generation between 0.3 - 0.5 µm and third generation between 0.5 - 0.7 µm. The distributions of experiments and simulations correlate very well. Relative intensities of the three generations as well as the absolute values are comparable. This concludes that the particles are indeed randomly distributed in the colloidal crystals. The lack of phase separation and prevention of heterogeneous film formation kinetics is a prerequisite for the characterization shown in this work.

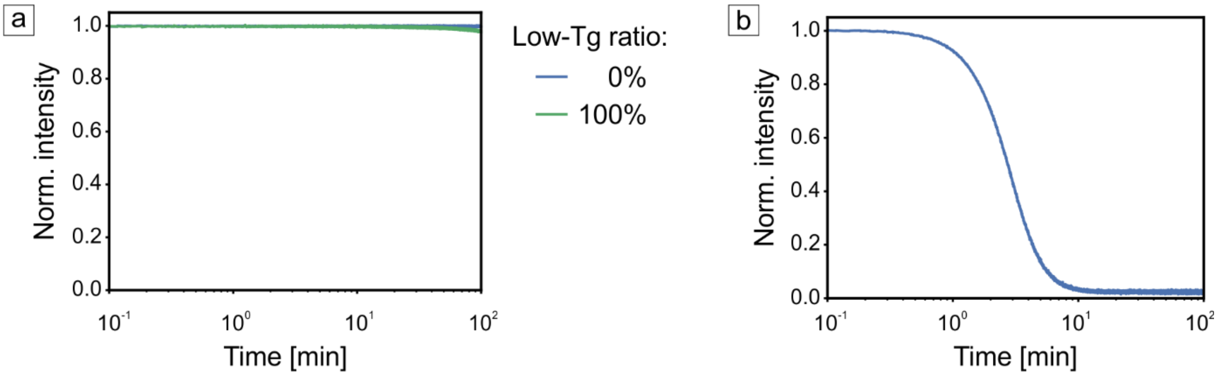

Figure S6. a) Stopband decay curves of colloidal crystals consisting purely of high- and low-T_g particles, respectively, at 45 °C. b) Stopband decay curve of 100% high-T_g particles at 100 °C. These measurements show the temperature limits accessible with the present combination of copolymer colloids.

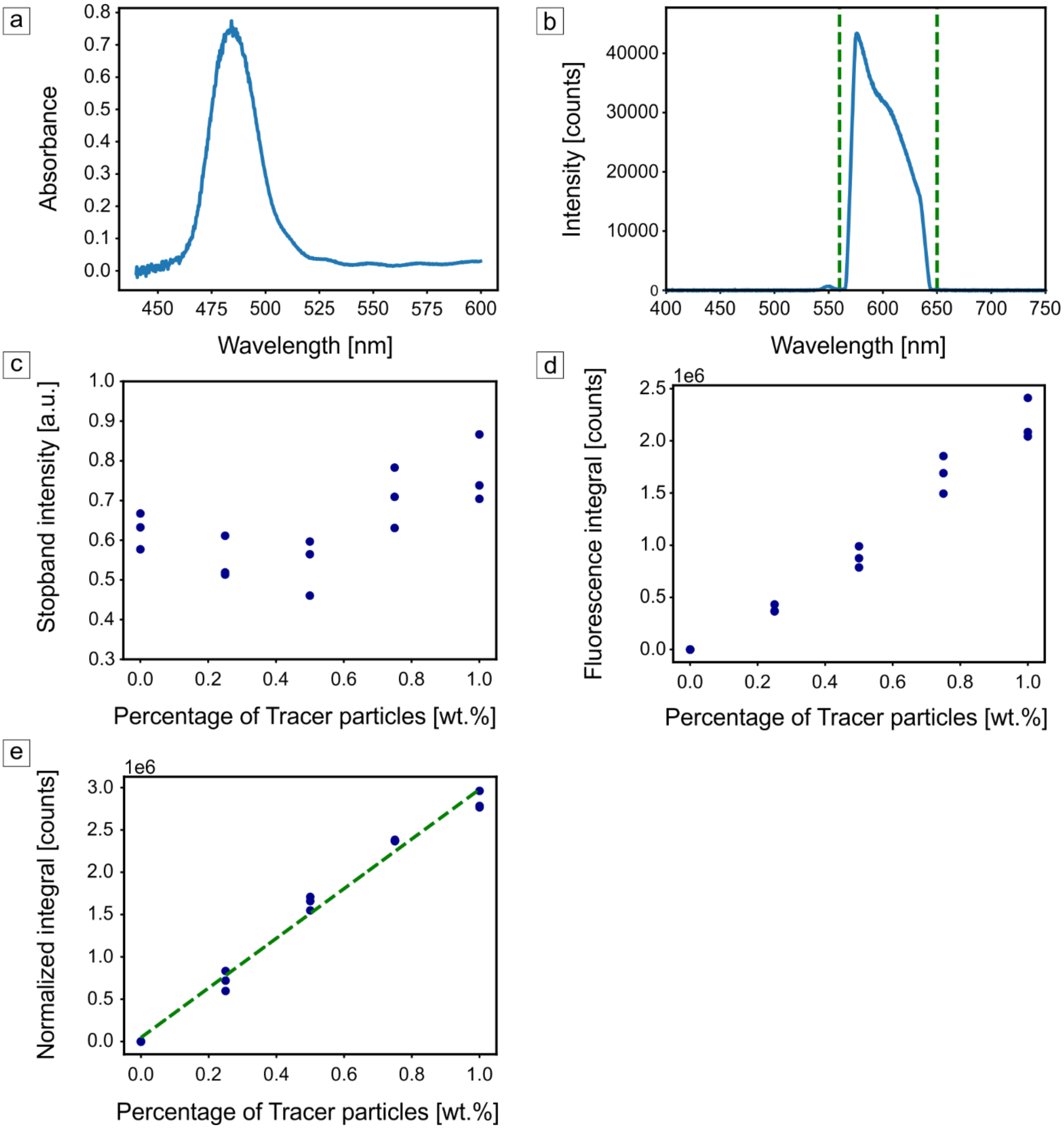

Figure S7. Exemplary a) baseline-corrected transmission UV-Vis and b) fluorescence emission spectra of a PMMA/nBA colloidal crystal prepared via dip-coating with 1.0 wt.% red-fluorescent polystyrene particles. Green lines in b) show the integration boundaries required for further evaluation. c) Determined stopband intensity and d) fluorescence integral of all measurements. e) Normalized fluorescence integral obtained for the various percentages of tracer particles. The green dotted line represents the linear fit used for the correlation of position and composition of the gradient in **Figure 3f**.

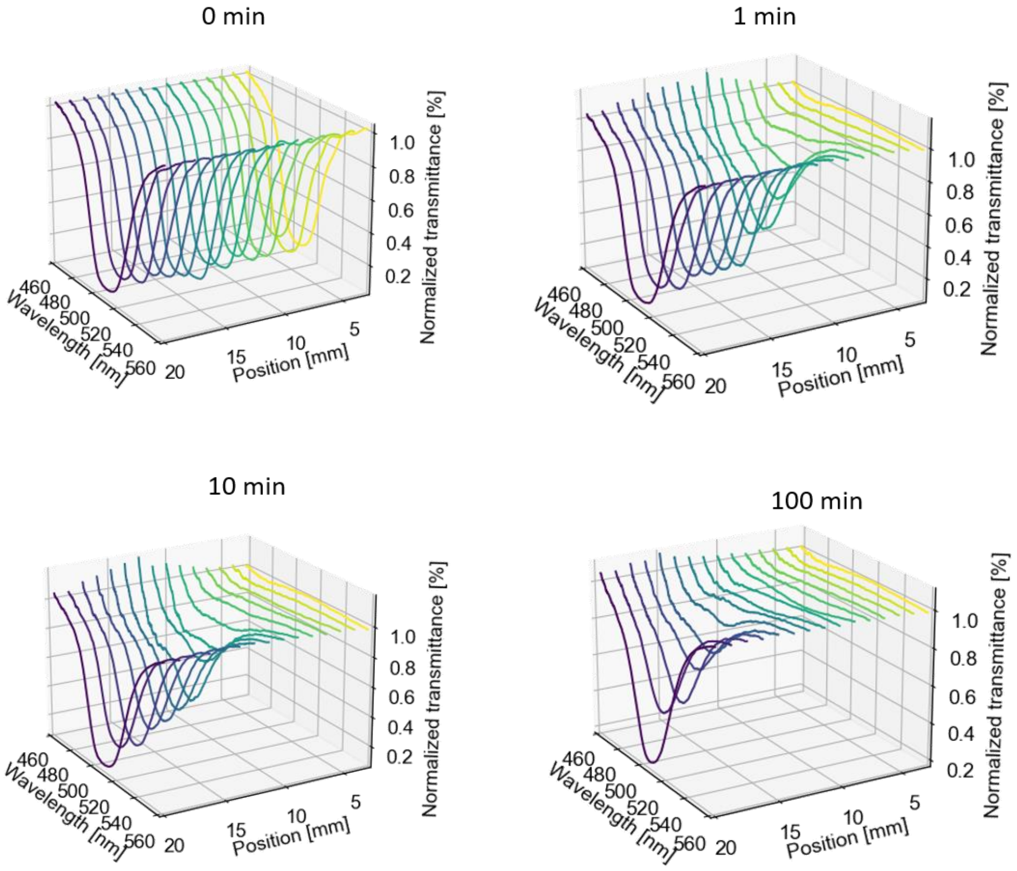

Figure S8. Baseline corrected transmission UV-Vis spectra measured *ex-situ* along a gradient colloidal crystal. These show the film formation process at 90 °C and how the degradation proceeds along the gradient.

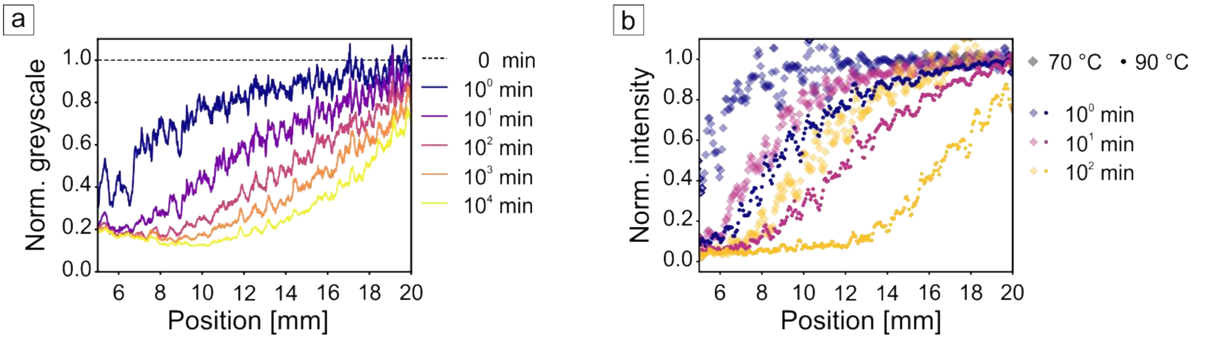

Figure S9. Measurements of the colloidal crystal gradient after different sintering times at 70 °C. a) Greyscale profile analysis *via* green channel separation of photographs shown in **Figure 4a**. b) *Ex-situ* UV-Vis results of a gradient sintered at 70 °C (large symbols) and 90 °C (small symbols) at equivalent sintering-times. The near overlap of the time-temperature pairs 100 min / 70 °C and 1 min / 90 °C shows the mutual influence of both time and temperature on the local film formation.

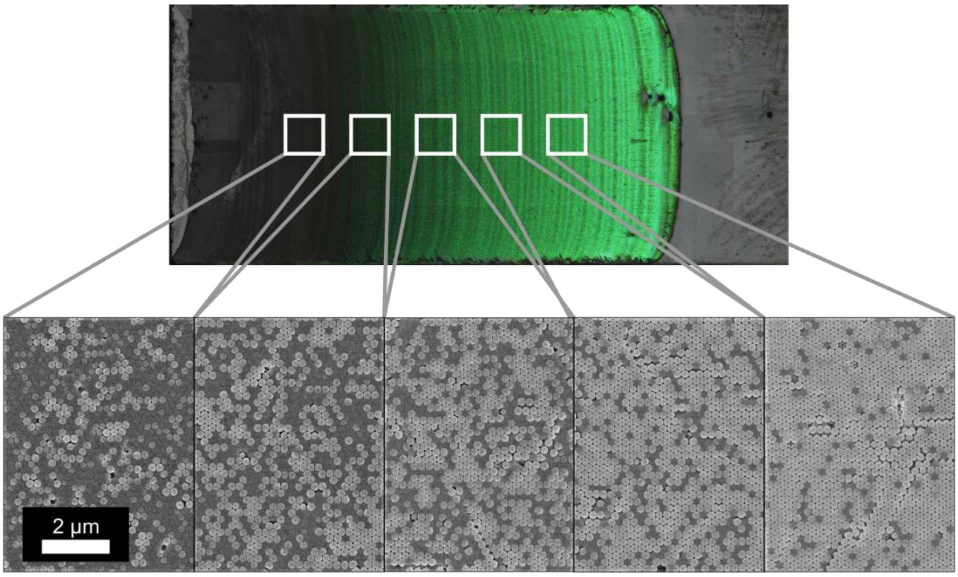

Figure S10. Scanning electron microscope images of a colloidal crystal gradient sintered at 70 °C for 100 minutes. A gradual decrease in the percentage of low-T_g particles is observed from left to right. The random particle distribution corresponds well to the individually prepared mixtures shown in **Figure S4**.

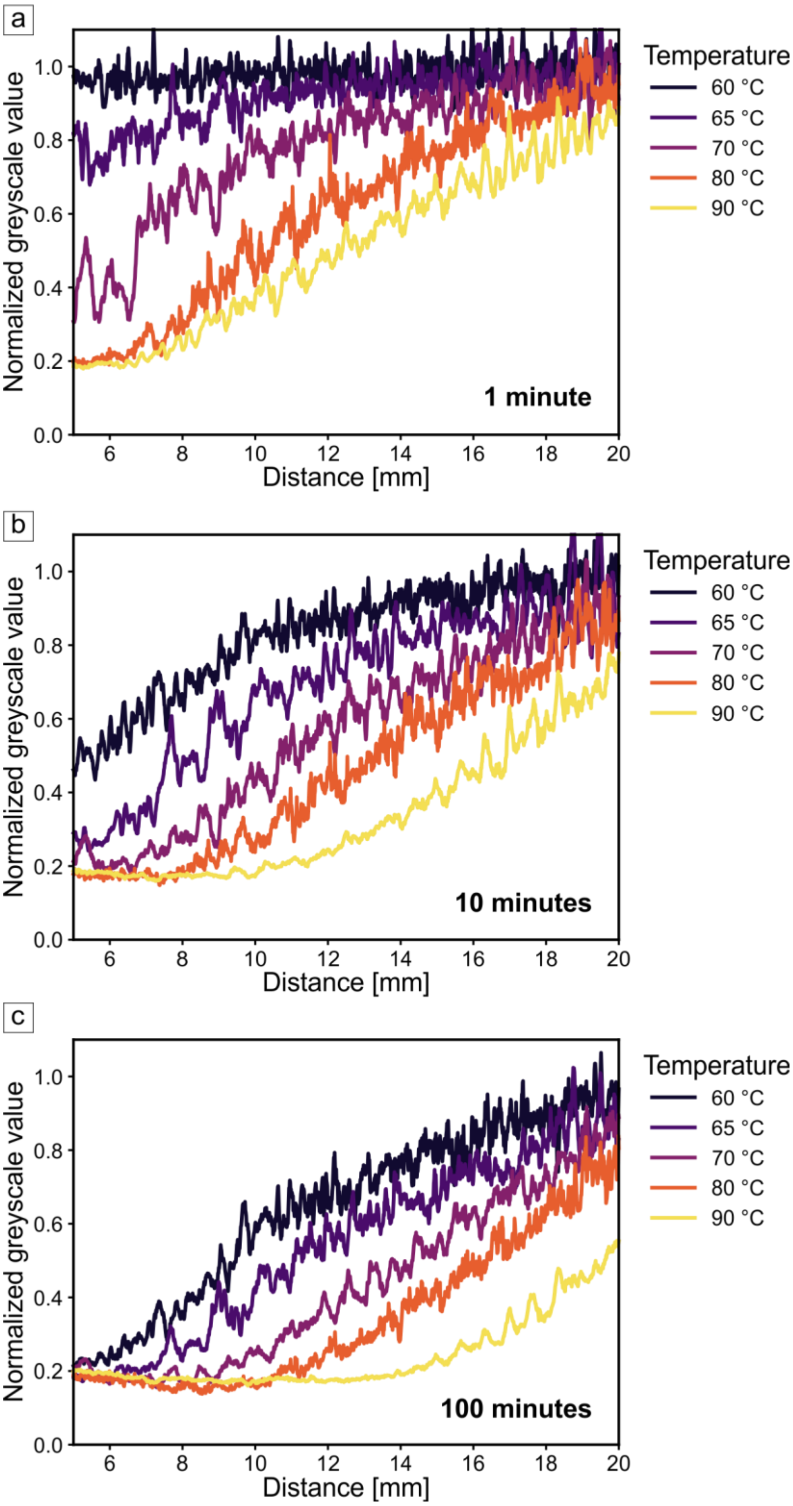

Figure S11. Temperature accuracy evaluation. Five gradient colloidal crystals are subjected to temperatures between 60 – 90 °C and the respective profiles are determined via green-channel image analysis. Three sintering times of a) 1 minute, b) 10 minutes and c) 100 minutes are presented. Temperature difference of 5 °C and higher can be distinguished.

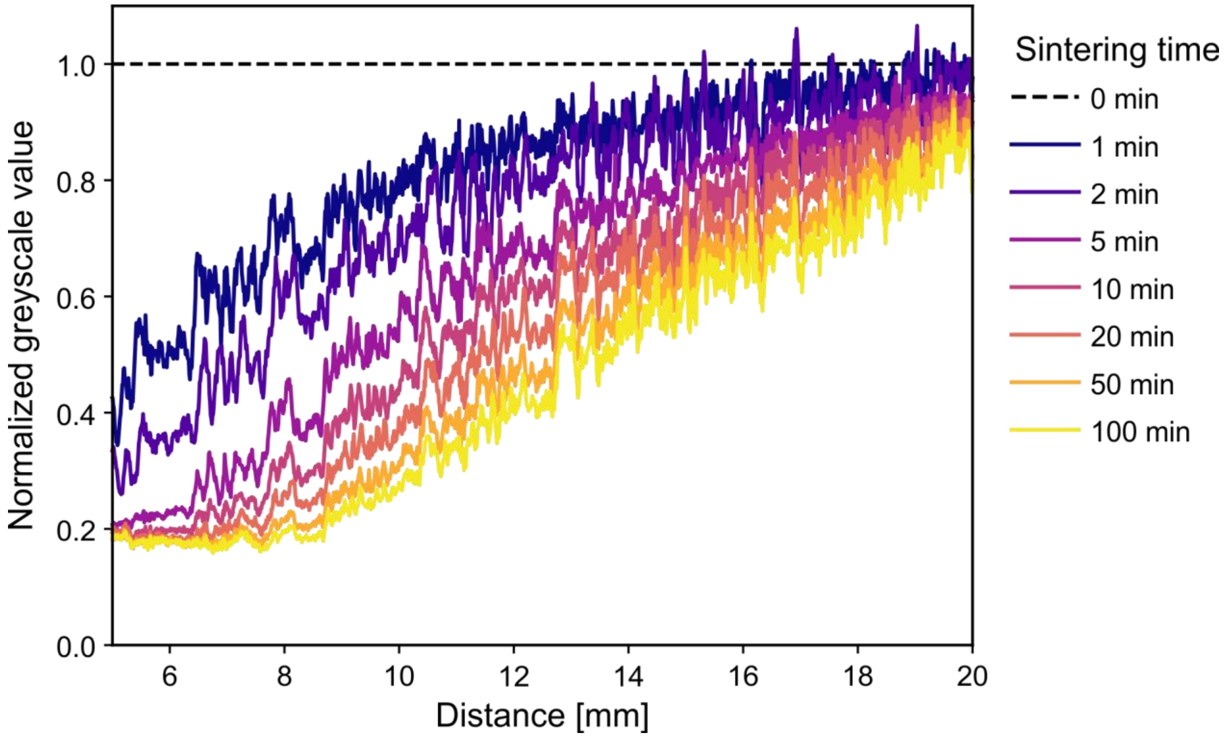

Figure S12. Time accuracy evaluation. A gradient colloidal crystal is subjected to a temperature of 70 °C and ex-situ profiles are obtained at various sintering times. The respective time decades (1 min, 10 min, 100 min) are well separated, inferring a temporal accuracy of a fraction of the sintering time of interest.

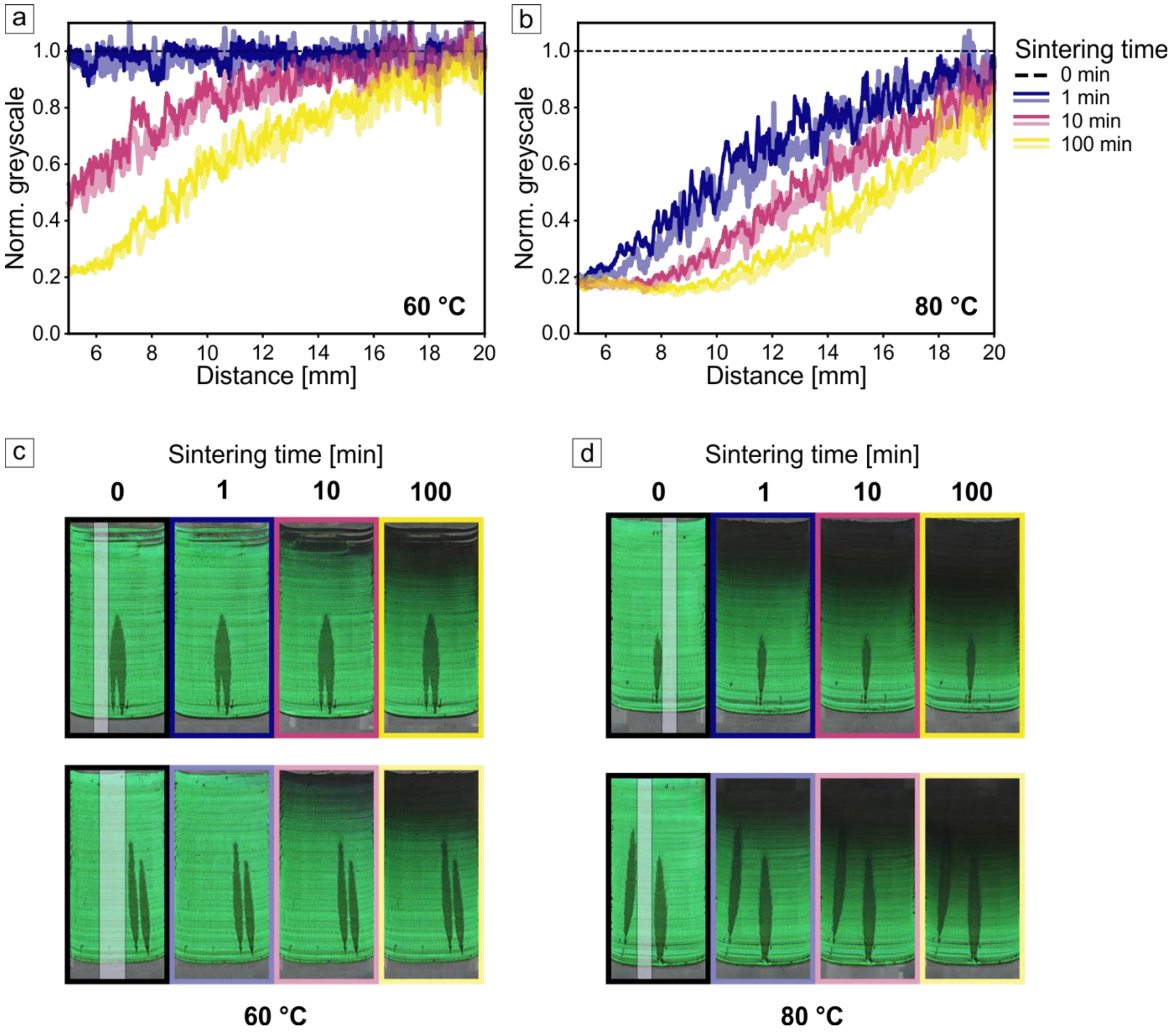

Figure S13. Reproducibility evaluation. Gradient colloidal crystals are subjected to sintering temperatures of a) 60 °C and b) 80 °C and profiles are determined via green-channel image analysis. Two samples are measured at each temperature and the resulting profiles are presented together (shown as an overlay of dark and light curve for each sintering time). The corresponding samples at the given sintering times are shown in c) and d), respectively. The high agreement between the two samples at both temperatures showcases the reproducibility of the time-temperature integrator. The green-channel profile analysis is conducted in regions of the samples (white shaded area) unaffected by the dark-green defects.
